# The co-administration of quetiapine or placebo to cognitive-behavior therapy in treatment refractory depression: A preliminary trial

**DOI:** 10.1186/1471-244X-8-73

**Published:** 2008-08-28

**Authors:** Yves Chaput, Annick Magnan, Alain Gendron

**Affiliations:** 1 Associate Professor of Psychiatry, McGill University Montreal, Quebec, Canada; 2 Assistant Professor of Psychiatry, University of Montreal, Montreal, Quebec, Canada; 3 "Centre de Psychologie René Laënnec", 1100, Beaumont Ave, Montreal, Quebec, H3P 3H5, Canada; 4 Neurosciences, Medical Affairs, AstraZeneca Canada Mississauga, Ontario, Canada; 5 Current address: 365 rue Normand, suite 230, Saint-Jean-sur-Richelieu, Quebec, J3A 1T6, Canada

## Abstract

**Background:**

Patients with major depression refractory to repeated pharmacological trials (TRD) may remain symptomatic for many years after their index episode. Augmentation strategies (with lithium or an atypical antipsychotic) or combining an antidepressant with short-term psychotherapy have been used with relative success in these patients. The aim of this study was to assess the effectiveness of the concomitant administration of quetiapine, an atypical antipsychotic, or placebo, to cognitive-behavior therapy (CBT) in TRD.

**Methods:**

Thirty-one patients who met entrance criteria for unipolar major depression (TRD stage II or greater) underwent 3 weeks of lithium augmentation after which non-responders (N = 22) were randomized to receive either quetiapine or placebo as an adjunct to their 12 weekly CBT sessions (quetiapine/CBT or placebo/CBT groups). Primary efficacy measures were the Hamilton and the Montgomery-Asberg rating scales for depression.

**Results:**

Overall, there was a significant reduction in both primary efficacy measure scores at LOCF for the 11 patients in the quetiapine/CBT group but not in the placebo/CBT treated patients. Patients in the quetiapine/CBT group, compared to those receiving placebo/CBT, showed a significantly greater degree of improvement on one primary and one secondary efficacy measure, were more likely to complete the trial and, completed a greater number of CBT sessions.

**Conclusion:**

Although preliminary, our results suggest that the adjunctive administration of quetiapine to CBT may prove useful in the treatment of stage II TRD.

**Trial Registration:**

Current Controlled Trials ISRCTN12638696.

## Background

Many depressed patients fail to respond to an adequate treatment with a single or, to several antidepressant trials, constituting what can be generally termed treatment refractory depression (TRD) [1]. The 'Sequenced Treatment Alternatives to Relieve Depression (STAR*D)' study has shown that only 50 to 55% of patients attain remission, defined by a Hamilton Rating Scale for Depression (HRSD) score of 7 or less, following the sequential administration of two antidepressant treatments [2].

The high degree of impairment and suffering imposed by TRD has led clinicians to search for an ever-increasing array of effective treatment strategies. Augmentation (with lithium carbonate or an atypical antipsychotic), switching to an antidepressant from a different class, combining several different antidepressants or an antidepressant with short-term psychotherapy, has been used with relative success in these patients [2-9]. Continued unresponsiveness despite persistent treatments has lead to the development of classification schemes for TRD [1,10]. Stage II TRD for instance is defined by the failure of two prior antidepressant treatments, each from a different class [1,10].

Quetiapine, an atypical antipsychotic with a broad spectrum of use including anti-anxiety and mood stabilizing properties, has proved useful as mono therapy in bipolar depression and, as adjunctive SSRI treatment in major depression [11-14]. As, by definition, patients in stage II TRD are refractory to the standard antidepressants, assessing quetiapine's potential benefit in these patients was thus of interest. Additionally, cognitive-behavior therapy (CBT) has been shown to result in outcomes 'generally comparable' to those of patients randomly assigned to alternative pharmacological strategies following an unsatisfactory response to prior treatment with the selective serotonin reuptake inhibitor (SSRI) citalopram [9]. This suggests that CBT may be of use in stage II TRD. Such a finding would not only be pertinent in TRD but also, in the treatment of depressed patients where medication may pose a health risk, such as during pregnancy or in the aged.

The aim of this study was to assess the effectiveness of the concomitant administration of quetiapine or placebo to patients receiving CBT in stage II (or greater) TRD.

## Methods

### Subjects

**Figure 1:**
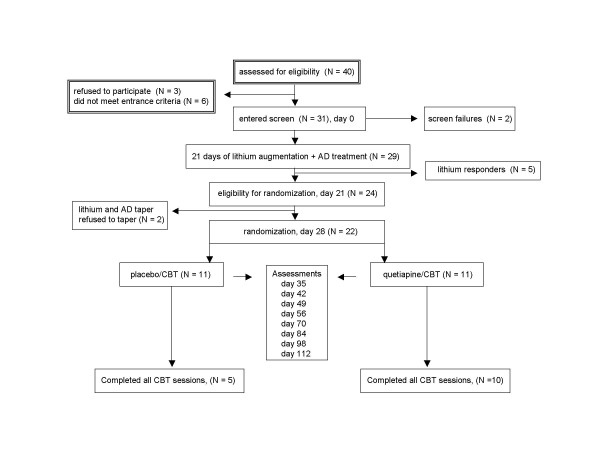
Study design and patient flow.

Study design and patient flow are depicted in Figure 1. Overall, 40 patients underwent a preliminary evaluation for entry into this study. Three signed informed consent forms but refused to enter into the screening phase of the trial whereas 6 were judged not to suffer from a major affective disorder and were excluded. The remaining 31 patients ranged in age from 23 to 66 and were recruited from the psychiatric outpatient service (N = 12), the psychiatric emergency service (N = 5), their family physicians (N = 12) or psychologists (N = 2). Patients met DSM-IV criteria for unipolar major depression with a HRSD (21 items [15]) score of ≥ 20 at screen and of ≥ 18 at both days 21 and 28 (randomization). A Clinical Global Impression severity scale (CGI-S, [16]) score of 4 or more at all three-evaluation points was also required.

Treatment refractoriness, verified by examining any pertinent medical records or charts, was determined by the failure of 2 (or more) 8-week treatments with 2 different classes of antidepressants. In addition, for at least 3 of these eight weeks, doses were required to be at or near the highest therapeutically recommended doses [17,18]. In 27 of the above patients venlafaxine was judged to have been sufficiently administered to constitute an adequate trial (225 mg/day, N = 19; 300 mg/day, N = 6; 375 mg/day N = 2). Citalopram was prescribed in 14 of these 27 patients (40 mg, N = 8, ≥ 50 mg, N = 6). Other individual antidepressants were prescribed much less frequently (N values from 1 to 5). Although 5 patients received sertraline and 5 paroxetine, 3 from each of these latter two groups received less that the highest recommended dose (150 rather than 200 mg and 40 rather than ≥ 50 mg, respectively). All other treatments (fluoxetine, mirtazapine, nefazodone, phenelzine, tricyclic antidepressants, moclobemide, bupropion) followed the standard dosing recommendations (Table 1, footnote *f*). Overall, these dose ranges were equivalent to those shown to induce remission (or response) in the STAR*D study [2].

**Table 1:** Patient demographic and clinical characteristics.

	**Q+CBT** **(N = 11)**	**P+CBT** **(N = 11)**	**All patients** **(N = 31)**
Women	8 (73%)	8 (73%)	23 (74%)
Average age	41.6 ± 13	44.9 ± 10	43.7 ± 11
Average # of treatments*^f^	3 ± 1	3 ± 1	3 ± 1
Average onset of depression	21 ± 15 mo	24 ± 15 mo	22.7 ± 16 mo
Prior augmentation **	4 (36%)	4 (36%)	11 (35%)
Prior ECT***	1	1	2
Mean HADS at screen	30 ± 6.4	29.8 ± 6.7	28.4 ± 6.7
Mean HRSD at screen	23.4 ± 3	22.4 ± 4.7	23 ± 3.5
Mean MADRS at screen	30.5 ± 3	29.8 ± 5	30.7 ± 4
Mean CGI at screen	4.2 ± 0.4	4.2 ± 0.4	4.2 ± 0.4

* Although patients had, on average, 3 adequate antidepressant trials (see ^*f*^) many also received antidepressants at lower dosages than those described below.

^*f *^Therapeutically recommended daily doses were; venlafaxine, 225 mg/350 mg (moderate, severe depression, respectively), citalopram 40 mg, sertraline 200 mg, mirtazapine 45 mg, nefazodone 600 mg, paroxetine 50 mg, amitriptyline 300 mg, phenelzine 90 mg [17,18].

** With either lithium carbonate or an atypical antipsychotic.

*** Electroconvulsive therapy.

Primary exclusion criteria included patients with a current risk of suicide, women of childbearing potential who were pregnant (or planning pregnancy), breast-feeding or not using medically adequate means of birth control for 3 months prior to admission into the study. Also excluded were patients with a DSM-IV diagnosis of bipolar disorder, schizophrenia, personality disorder (borderline, antisocial, schizoid, schizotypal or paranoid), panic, generalized anxiety, obsessive-compulsive, somatoform or organic mental disorder, anorexia nervosa, bulimia or those with definite or suspected substance abuse in the previous 12 months. Although exclusion was primarily based upon the clinical interview, whenever possible, supplementary medical documentation, including hospital charts, were reviewed. Patients requiring concurrent treatment with any psychotropic medication other that that permitted in the trial (zopiclone or temazepam as hypnotics on a PRN basis) or those with serious or unstable medical illnesses, known psychotropic drug allergies or co-existing diseases or treatments that might contraindicate the use of the study drug were also excluded.

The study was conducted at 2 sites in close proximity to one another (by the same investigators) in Canada and was approved by both a University and an Independent Institutional Review Board. After the study was completely described to the patients written informed consent was obtained in accordance with the Helsinki Declaration.

### Study design

Patients underwent a screen evaluation where a complete psychiatric history, including previous and current pharmacological therapy, was obtained. A physical examination was performed along with an electrocardiogram. Weight, height, blood pressure (sitting and standing) and pulse were recorded and blood samples were drawn for routine blood biochemistry (liver enzymes, creatinine, electrolytes, calcium, glucose and prolactin), hematology (hemoglobin, hematocrit, RBC, WBC, differential and platelets) and endocrinology (TSH, serum pregnancy test). A urine sample for analysis and drug screen was taken.

Evaluator-rated scales at screen included the HRSD and the Montgomery-Asberg Depression Rating Scale (MADRS, [19]), which constituted the primary efficacy measures. Secondary efficacy measures at screen included the investigator-rated Clinical Global Impression, severity and improvement (CGI-S, CGI-I), the Extra-pyramidal Symptom Rating Scale (ESRS, [20]) and the Barnes' Akathisia Rating scale (BAS, [21]). Patient-rated secondary efficacy measures at screen were the Hospital Anxiety and Depression Scale (HADS, [22]) and the Quality of Life Enjoyment and Satisfaction Questionnaire (Q-LES-Q, 16 question version, [23]).

Patients who continued to meet study entrance criteria underwent an initial 3-week open phase of lithium augmentation (≥ 600 mg per day, serum levels of between 0.6 and 0.9 mEq/L by day 7) of their antidepressant treatment (using therapeutically recommended doses). HRSD scores were reassessed at day 21 and patients with a ≥ 40% reduction (or a score < 18) were classified as responders and excluded. Non-responders were tapered of their medication (lithium and antidepressant) during an 8-day period and reassessed for randomization at day 28 (week 4). Those with a HRSD of ≥ 18 were randomized to one of two treatment groups; placebo and CBT (placebo/CBT) or quetiapine and CBT (quetiapine/CBT).

### Randomization

Subjects were randomized strictly sequentially. If a subject discontinued from the study, the subject number was not reused, and the subject was not allowed to re-enter the study. Individual treatment codes were in sealed envelopes (code-breaks, indicated the treatment allocation corresponding to a blinded drug blister pack) for each randomized patient. These were provided by AstraZeneca IPS(UK). AstraZeneca Canada Inc kept one set of code envelopes. The investigator kept a second set.

### Treatment

CBT was administered in 12 weekly ≥ one-hour sessions given in an individual setting by the same therapist (an MSc level psychologist with over 7 years of clinical experience supervised by a PhD level psychologist specialized in CBT). Individual, rather than group CBT treatment was chosen as there is evidence to suggest that the latter may be less efficacious than individually administered CBT in alleviating depressive symptoms [24]. The CBT paradigm, modified from that used in a previous study [24], was largely based upon the Beck cognitive therapy model with its associated behavioral techniques [25]. In addition, social skills training and applied relaxation training was also administered [26,27]. Homework assignments were, whenever possible kept in record form and monitored by the therapist at the following session.

Medication was administered orally with an initial dose of 12.5 mg for quetiapine and placebo, twice daily. Quetiapine was titrated up to a maximum of 200 mg twice daily within the first 14 days following randomization using a flexible dose schedule dependent upon the patient's clinical response and the side-effects profile. The dose remained stable thereafter. Compliance was monitored by capsule counts of returned medication at each visit.

### Assessments

Safety (vital signs, weight, adverse events) assessments were performed weekly up to week 8 (day 56) and once every 2 weeks thereafter. Electrocardiograms were repeated at randomization (week 4) and midway through the active phase of the study (week 10). Hematology, biochemistry, and urine analysis were repeated at the final visit (week 16). The following rating instruments (HRSD, MADRS, CGI-S and CGI-I) were performed again at weeks 3 and 4 and at every two weeks thereafter. The EPS scale, the BAS, the HADS and Q-LES-Q were repeated at week 16 or at LOCF following randomization.

### Statistical analysis

The statistical analysis package Systat (Version 11) was used for power and, for statistical analysis. Given the study population the trial was designed to detect a moderate effect, such as a 5 to 6 point reduction of the HRSD. Several scenarios were run with initial HRSD scores of between 20 and 25 at screen, scores not unreasonable for this type of population. Results suggested that a minimum of 9 to 10 patients randomized to each treatment group were needed in order to yield sufficient power (80% to 90%) to detect a within group effect (alpha set at 0.05).

The safety analyzable population was defined as all patients who received at least one dose of either lithium carbonate (N = 30) or of study drug (N = 22). The intent-to-treat population (ITT), used for efficacy analyses, consisted in all baseline patients who were randomized and took at least one dose of study drug and who had at least one post-randomization assessment (N = 22). All efficacy analyses used either last observation carried forward (LOCF) or data on patients available at each study visit. Baseline comparability of the two treatment groups for demographic and clinical variables was assessed by two-sample *t*-tests for continuous variables and the X^2 ^test for categorical variables. Response was defined as above (HRSD score reduction of ≥ 40% or a score of < 18). Within group efficacy analyses were performed using two-sample *t*-tests for paired variables. Between group efficacy analyses were performed using analysis of covariance (ANCOVA) using change from baseline as the dependent variable and baseline measurements as the covariate.

## Results

### Lithium carbonate administration in TRD

Clinical and demographic variables for the screened and for the randomized patients are detailed in Table 1. Of note is a prior history of electroconvulsive treatment (2 patients, one in each randomized group) suggesting that some patients had greater than stage II refractory depression. Patient sub-samples were not significantly different with regards sex, age, duration of illness, prior antidepressant treatment (for the current episode) and initial rating scale scores from the pooled values of the 31 patients initially screened for the study. Of the 31 patients screened, one was withdrawn prior to the lithium augmentation phase due to a urine test positive for drugs of abuse. A second patient was withdrawn during the augmentation phase due to lithium non-compliance, leaving 29 completers of this open phase of the study. Of these latter patients 5 (17%) were classified as lithium responders (HRSD of 23 ± 4 at screen, 12.4 ± 2.3 at day 21) and were subsequently withdrawn from the study. Two patients classified as lithium non-responders withdrew consent no longer wishing to taper their concomitant antidepressant treatment. Twenty-two patients were thus randomized to the CBT portion of the trial.

### Quetiapine and CBT administration versus placebo and CBT in TRD

Primary efficacy measures were significantly (p < 0.05, *t*-test for paired values) improved in patients randomized to quetiapine/CBT (HRSD 23.5 ± 3 at randomization, 16 ± 5 at LOCF; MADRS of 30.5 ± 3 at randomization, 22 ± 7 at LOCF). In contrast, no significant improvement in was observed in the placebo/CBT group. Results for the secondary efficacy measures mimicked those of the primary efficacy measures, as shown in Table 2. Only two of the four secondary efficacy measures (CGI-S and CGI-I) showed statistical improvement in the placebo/CBT group.

**Table 2:** Percent improvement of secondary rating scale scores

	**Q+CBT** **(N = 11)**	**P+CBT** **(N = 11)**	**Between group ** **differences**
HADS	20%^a^	6%	ns
CGI-S	33%^c^	12%^a^	ns
CGI-I	33%^c^	23%^a^	ns
Q-LES-Q	29%^b^	2%	p < 0.05^d^

^a ^p < 0.05, *t*-test for paired values comparing screen (or randomization) and LOCF scores for individual rating scales within each group.

^b ^p < 0.01, *t*-test for paired values comparing screen and LOCF scores for the Q-LES-Q rating scale within each group.

^c ^p < 0.005, *t*-test for paired values comparing screen (or randomization) and LOCF scores for individual rating scales within each group.

^d ^ANCOVA using screen scores as covariates and LOCF scores as outcome.

Significant between group differences (ANCOVA using baseline rating scores as covariates and LOCF scores as outcome) were observed between the two treatment groups with regards one primary (HRSD, p < 0.05) and one secondary efficacy measure (Table 2). An interesting between group observation was study completion (the number of patients completing the study versus the number of dropouts). Ten of 11 patients in the quetiapine/CBT group completed the trial whereas 5 of 11 completed the study in the placebo/CBT group. This was reflected in a significant difference in the number of completed CBT sessions between the 2 groups (p < 0.05, ANOVA, mean number for the quetiapine/CBT group was 11 ± 2, the mean for the placebo/CBT group was 7 ± 5). Of note is that the 5-placebo/CBT completers showed a statistically significant reduction in both primary rating scale scores at LOCF (HRSD 22 ± 1.4 versus 15 ± 2.3 p < 0.01, MADRS 31 ± 2.6 versus 19 ± 2.4, p < 0.01, *t*-test for paired values).

The average dose of quetiapine attained within the first 14 days following randomization was lower than that typically used in the treatment of chronic psychosis (147.7 ± 112 mg/day, range of 25 to 375 mg). Patients in the placebo/CBT group had a slightly higher average dose (209.1 ± 120 mg/day, range 50 to 400 mg), although the difference was not statistically significant. The average number of pills per day for both groups was similar (1.9 ± 0,6 pills per day for quetiapine/CBT, 2.1 ± 0,7 for placebo/CBT). Quetiapine was well tolerated with an adverse event profile from mild to moderate. A non-significant increase in body weight was observed in both groups. No serious adverse events were observed and no patient withdrew due to a drug-induced event. One patient (quetiapine group) was withdrawn due to a possible anomaly detected at the week 10 electrocardiogram that was ultimately found to be a false positive result. Common quetiapine(n)/placebo(n) related adverse events were somnolence (7/1), insomnia (5/2), headache (4/1), dry mouth (4/1), nausea (2/2) gastrointestinal discomfort (2/3) and labile hypertension (1/1). Only somnolence was found to be statistically significant between treatment groups (p < 0.01, Fisher's exact test). Although one patient (placebo group) was observed to suffer from mild akathesia and muscle rigidity, overall, no significant changes were seen in the BAS or EPSS scales in either group at LOCF.

## Discussion

Our results suggest that quetiapine/CBT is more effective that placebo/CBT in TRD. Although preliminary, these results are compatible with both clinical and pre-clinical evidence suggesting that quetiapine is a broad spectrum antipsychotic potentially useful in treating bipolar (in mono therapy form) and unipolar (as an adjunct to a standard SSRI) depression [11-14]. Whether quetiapine mono therapy would have yielded similar results remains to be determined. In this trial it was solely administered as an adjunct to CBT and CBT (mono therapy or combined with pharmacotherapy) has been successfully used in uncomplicated depression, residual and chronic depression [9,28-31]. In the present study, placebo/CBT patients had a very high dropout rate. Completers however showed reductions in primary rating scale scores equal to those in the quetiapine/CBT group.

The average percent reductions of both primary rating scale scores in the quetiapine/CBT group, although significant, were admittedly modest (30%). Although a different psychotherapeutic modality was used, Keller et al., [28] reported a 73% response rate (remission and satisfactory response) for the combined psychotherapy/antidepressant treatment of 'chronic' depression. However, those with a satisfactory response only (a HRSD ≤ 15) represented about half of these patients and overall, 16 to 20 CBT-analysis system sessions were administered [28].

The modest rates reported here can be explained in several ways. First, a lower overall (17%) response rate to lithium augmentation was also observed. Interestingly, although this rate is substantially lower than that typically reported for lithium augmentation in patients following single pharmacological trials [3,4] it was nevertheless almost identical to that reported for patients not responding to several prior antidepressant treatments [32]. It is therefore possible that the modest results achieved by both active treatments in this study (lithium, quetiapine/CBT) are partially related to the high number of prior pharmacological trials our patient sample received. Second, this study was a preliminary one and was designed to measure response, rather than maximal response or remission. It is possible that the number of CBT sessions administered (12 sessions) and the time frame used in the lithium augmentation phase (3 weeks) of the study were suboptimal. Shapiro et al., [33] observed no significant difference between an 8 and a 16 week CBT protocol in the treatment of depression. However, a meta-analytic review of several short-term psychotherapies for depression suggests that a minimum of 13 sessions may be required in order to approach what might be termed maximum benefit [31]. In addition, although lithium augmentation can be efficacious within 48 hours of its administration [4] delayed responses (up to 8 weeks) have also been frequently observed [3]. Therefore, longer durations of both lithium and quetiapine/CBT treatments might have resulted in a greater number of patients responding. It may also have revealed more improvement in the placebo/CBT group. In this same light, the present study used a flexible dose schedule of quetiapine that was in part based upon patient input. In contrast to the standard antidepressant drugs, a recommended 'effective' quetiapine dose was simply not available. Therefore, although our average dose approximated that found to be efficacious as an adjunct to SSRI treatment in non-bipolar depression [13] it was much lower than the mono therapy fixed-doses used to treat bipolar depression [12]. Higher doses may have resulted in a greater response. Indeed, these methodological considerations represent one of this study's main limitations.

Third, entrance criteria for the present study were relatively stringent and should have helped reduce the placebo-expectancy response rate [34]. Stage II TRD was the baseline although some patients were closer to stage V [1,10]. Patients had an average HRSD score ≥ 22 at both screen and randomization (28 days later). An effort was made to eliminate patients with severe or obvious co-morbid psychiatric diagnoses. In addition, the time course of this trial was quite long. For instance, there was a minimum 28-day period from screen to randomization. More typically, up to an additional week was required in order to verify all previous medication. Patients were seen and assessed weekly during the open label phase of the trial. As such, any possible early, non-specific therapeutic effect produced by this intensive follow-up might have dissipated by randomization (primary rating scale scores for the 22 randomized patients were almost identical at screen and randomization). These patients may have thus represented a more homogeneous (or endogenous) group than those typically reported in CBT studies of depression and chronic depression [9,28,31]. Although the relationship between the efficacy of CBT and the initial severity of the depressive syndrome (inversely correlated) is still unclear [30,33,35,36], such a phenomenon may have also contributed to the overall low level of response, especially in the placebo/CBT arm of the study.

The results of clinical trials where a much greater number of patients were treated suggest that a combination 'pharmacotherapy/psychotherapy' approach may be superior to either treatment alone (or with placebo) in difficult to treat depression [9,28]. Our data neither contradicts this contention nor supports it. Assessing the antidepressant effectiveness of quetiapine mono therapy in similarly ill patients would provide a more definitive answer. Our results, as well as the increasing number of off-label indications for quetiapine [11,12,14,37] would certainly provide a reasonable premise for such an undertaking. Quetiapine (but not haloperidol) has been shown to improved cognitive skills in chronic psychotic patients [38]. Therefore, factors other than (or in addition to) an antidepressant effect might also have contributed to quetiapine's effectiveness in our study. These could also include non-specific sedative effects that might have influenced our results by helping to stabilize patients during the antidepressant withdrawal phase, better permitting them to focus on the tasks at hand. The absence of this effect in the placebo/CBT group may have contributed to the high dropout rate and, as a consequence, the relatively poor improvement in this group. This may have been at least partially responsible for the very low dropout rate and the better performance of the combined treatment group.

## Conclusion

The relatively small number of patients in this trail as well as the many methodological considerations mentioned above precludes a broad generalization of our results. Nevertheless, we provide preliminary evidence that combined quetiapine/CBT treatment may offer a clinically pertinent alternative for patients with stage II TRD. This finding is consistent with evidence suggesting that both treatment modalities are evolving into broad spectrum strategies useful in the treatment of pathologies ranging from anxiety, personality and eating disorders, uncomplicated, chronic or bipolar depression and schizophrenia [9,24,28,29,39-41].

## Abbreviations

TRD: Treatment refractory depression; CBT: cognitive-behavior therapy; ITT: intent to treat; LOCF: last observation carried forward; HRSD: Hamilton Rating Scale for Depression; MADRS: Montgomery-Asberg Depression Rating Scale; CGI-S: Clinical Global Impression – severity; CGI-I: Clinical Global Impression – improvement; HADS: Hospital Anxiety and Depression Scale; Q-LES-Q: Quality of Life Enjoyment and Satisfaction Questionnaire; EPS: Extra Pyramidal Symptom Rating Scale; BAS: Barnes' Akathisia Rating Scale; AD: antidepressant; ANCOVA: analysis of covariance.

## Competing interests

The authors declare that they have no competing interests.

## Authors' contributions

YC as primary author, was responsible for the development of the protocol, data and statistical analysis and, the writing of the manuscript.  AM had significant input as to the CBT protocol and was responsible for carrying out all cognitive behavior treatments.  AG was instrumental in piloting the protocol through AstraZeneca Canada, obtaining funding and in organizing most of the infrastructure of this trial.

## Pre-publication history

The pre-publication history for this paper can be accessed here:


